# Platelet-Rich Plasma and Electrochemical Biosensors: A Novel Approach to Ovarian Function Evaluation and Diagnostics

**DOI:** 10.3390/ijms26052317

**Published:** 2025-03-05

**Authors:** Tatjana Ivaskiene, Greta Kaspute, Egle Bareikiene, Urte Prentice

**Affiliations:** 1 State Research Institute Centre for Innovative Medicine, Santariskiu St. 5, LT-08410 Vilnius, Lithuania; tatjana.ivaskiene@imcentras.lt (T.I.); greta.kaspute@ftmc.lt (G.K.);; 2 Department of Nanotechnology, State Research Institute Center for Physical Sciences and Technology (FTMC), Sauletekio Av. 3, LT-10257 Vilnius, Lithuania; 3 Department of Physical Chemistry, Institute of Chemistry, Faculty of Chemistry and Geosciences, Vilnius University, Naugarduko St. 24, LT-03225 Vilnius, Lithuania; 4 Department of Mechatronics, Robotics and Digital Manufacturing, Faculty of Mechanics, Vilnius Gediminas Technical University, Plytines St. 25, LT-10105 Vilnius, Lithuania

**Keywords:** platelet-rich plasma, biomarkers, electrochemistry, molecularly imprinted polymers, diagnosis, prognosis, ovarian

## Abstract

Preserving ovarian function is important to women’s reproductive health. It is necessary for fertility and maintaining the overall hormonal balance. Platelet-rich plasma (PRP) is an autologous plasma containing a predominately platelet concentrate prepared from fresh blood. It has been observed that PRP injections into the ovary can renew the functional cells of the cortical layer of the ovary follicles and reactivate the production of sex hormones. It may improve a woman’s fertility in the case of premature ovarian failure, the condition after chemotherapy treatment, or during the climacteric period. The main markers to evaluate the procedure’s success are elevated anti-Müllerin hormone and enlarged count level of atrial follicles in ovaries. The aim of this review is to identify the ovarian PRP procedure success markers and point out the electrochemical sensor techniques. Literature was selected depending on including and excluding criteria; studies were sorted by topics in two blocks: PRP biomarkers and electrochemistry. As PRP acts as a regenerative care, electrochemical biosensors can provide accurate, real-time data to evaluate the biological response to PRP therapy. The biosensors’ ability to monitor hormonal levels and follicle development serves as objective markers of the effectiveness of PRP in restoring ovarian function. Together, these approaches enable a more precise evaluation of ovarian health and fertility outcomes after PRP intervention.

## 1. Introduction

The main function of the ovaries is to mature the follicles in the cortical layer, which synthesize sex hormones in their cells to maintain the body’s homeostasis and to form the oocyte, the female sex cell [1]. With age, the ovaries release fewer hormones; follicles do not mature. The main hormones defining ovarian function are oestradiol (E2), luteinizing hormone (LH) and anti-Müllerin hormone (AMH). At the same time, the hypothalamus synthesizes more gonadotropin follicle-stimulating hormone (FSH), which aims to stimulate the ovaries. As the ovarian function declines, the hormones synthesized in the ovary decrease in the body while the hypothalamic FSH hormone increases [2,3]. This leads to impaired reproductive health, perimenopausal symptoms (irregular menstrual cycles, hot flashes, sleep disturbances, mood swings, emotional changes, vaginal dryness) and the onset of the climacteric period [4]. The European Society of Reproductive Medicine (ESHRE) guidelines define premature ovarian insufficiency (POI) as the presence of oligomenorrhea–amenorrhea for at least 4 months [5] and serum follicle-stimulating hormone (FSH) levels of  ≥25 IU/mL measured at least twice with a 4-week interval, with an onset before the age of 40 years [5,6]. The available treatment options for POI are either to maximize the ovarian response from the available limited follicular pool or oocyte donation [7].

Platelet-rich plasma (PRP) is an autologous plasma containing a predominately platelet concentrate prepared from fresh blood. The number of platelets in such plasma is supraphysiological, three to five times higher than the physiological amount [8]. The PRP material also contains three blood proteins that act as cell adhesion molecules: fibrin, fibronectin, and vitronectin [9]. PRP injections are a modern medical method that can often help people regenerate tissues [10]. Most of the time, the ovarian PRP procedure is performed on an outpatient basis. The ovarian PRP procedure for women is performed under intravenous anaesthesia and takes up to 30 min [11].

Information transmission, which can be used to evaluate the success of analyte determination procedures in biological systems, is primarily electrochemical [12]. Electrochemistry in biological systems alludes to the movement and exchange of charged particles, such as ions and electrons, which are fundamental at the cellular level [13]. By coupling biochemical reactions with electrochemical mechanisms, biological systems achieve efficient energy transfer, precise signal transmission, and dynamic control of cellular processes [14].

Electrochemistry is particularly well suited for analysing biomedical samples due to its high sensitivity, fast response, and ability to perform real-time monitoring of key biomarkers [15]. PRP contains various growth factors, cytokines, and other molecules essential for evaluating its therapeutic potential [16]. Electrochemical sensors can detect these molecules at very low concentrations, ensuring accurate assessments [17]. Additionally, these sensors are cost-effective and require minimal sample preparation, making them accessible for routine analysis [18]. Cost-effectiveness also comes from materials used to prepare surfaces for biosensing applications. Nowadays, electrodes might be screen-printed, which makes the manufacturing process economically optimal.

Furthermore, electrochemical sensors can be miniaturized for portable, point-of-care applications, offering convenience in clinical and research environments [19]. Their real-time monitoring of PRP therapy allows clinicians to optimize treatment protocols and improve diagnostic efficiency. These advantages position electrochemical methods as a cutting-edge PRP analysis and regenerative medicine diagnostics approach.

An electrochemical sensor is a standalone device that converts electrochemical reactions into analysis-ready signals. It provides specific quantitative or semi-quantitative data using a chemical recognition element (receptor) integrated with the electrode straight [20]. The most used configurations for such sensors are the two-electrode (2E) and three-electrode (3E) systems [21]. Electrochemical sensors are well-suited for biomedical analysis [22].

This review uniquely integrates PRP and electrochemical biosensors for ovarian function evaluation, unlike previous studies that examined them separately. It offers an updated perspective by incorporating recent innovations and clinical applications. Electrochemical biosensors present a promising tool for ovarian function assessment due to their high sensitivity, rapid detection, and cost-effectiveness. Their capability for real-time, point-of-care diagnostics makes them a valuable alternative to traditional methods, expanding possibilities for reproductive health monitoring. This approach introduces a novel detection strategy, as limited research has been conducted in this field.

## 2. PRP Procedure: Possible Therapeutic Effects and Challenges

PRP procedure has gained significant attention in regenerative medicine. The procedure involves the patient’s own blood, which will be centrifuged and prepared for the autologous injection. The stars of this procedure are thrombocytes. Thrombocytes are cytoplasmic fragments of megakaryocytes formed in bone marrow. They are about 2 μm in diameter and contain membrane, cytoplasm, granules, and mitochondrial DNA [23]. Platelets contain more than 30 biologically active proteins, many of which play a key role in maintaining haemostasis or tissue growth and regeneration. Seven major protein growth factors actively secreted by platelets initiate wound healing (Table 1) [24,25].

A gynaecologist evaluates the changes in ovarian reserve to determine the results of the ovarian PRP procedure. It is evaluated by performing blood tests and ultrasound examinations before and after the procedure. Blood tests mostly include FSH and AMH. Ultrasound examination includes counting antral follicles (about 2–9 mm in diameter) [31]. Antral follicles are also referred to as resting follicles. The number of antral follicles visible on ultrasound indicates the number of microscopic primordial follicles remaining in the ovary [32]. Each primordial follicle contains an immature egg that can potentially develop and ovulate in the future [31]. As women age, they have less primordial and antral follicles remaining. When there are less than four antral follicles in both ovaries, ovarian insufficiency is diagnosed [31]; this is the part where the ovarian PRP procedure may help regenerate ovarian tissue and promote more atrial follicles to grow [33].

A systematic review and meta-analysis included 14 studies with 1632 participants (10 studies included women with POR, 1 included women with POI, and 3 studies included both POR and POI women) [1,8]. The results determined elevated ovarian function. AMH level was evaluated in 11 studies (2099 women), and antral follicular count (AFC) level was assessed in 6 studies (1399 women) [1]. The number of oocytes retrieved was evaluated in seven studies (1413 women) [1]. The conclusion of the systematic review showed a significant improvement in AFC, the number of retrieved oocytes, the number of cleavage embryos, and the cancellation rate in women with POR [1]. Another systematic review, published in 2024, assessed currently available articles on the PRP procedure and changes in ovarian function. A total of 38 studies involving 2256 women were assessed. A consistent trend was observed: AMH levels increased, FSH levels significantly decreased, and the number of antral follicles increased significantly after ovarian PRP treatment. A significant increase in the number of retrieved oocytes was observed. The spontaneous pregnancy rate after ovarian PRP treatment was 0.07, the biochemical pregnancy rate was 0.18, and the live birth rate was 0.11 times higher compared to women who did not undergo ovarian the PRP procedure. PRP resulted in a statistically significant improvement in key parameters of fertility in women with reduced ovarian reserve [34]. A retrospective study was conducted to determine the effect of platelet-rich plasma (PRP) injection on ovarian stimulation outcomes in women referred to IVF centres. Changes in ovarian hormones (FSH, AMH, E2, and LH) were evaluated before and after the procedure. A total of 469 women participated in the study. FSH levels were significantly reduced after PRP treatment in the largest cohort evaluating the efficacy of an intraovarian infusion of PRP for ovarian rejuvenation. The benefits were evident in all age groups studied. It was also found that autologous PRP infusion could restore ovarian function by activating the folliculogenesis process and enhancing the hormonal profile. This can help restore fertility [35]. Another study investigated the effect of a 3-month course of intraovarian injections of autologous platelet-rich plasma (PRP) on ovarian reserve markers, comparing women who received PRP injections with those who did not [36]. A total of 83 women participated in the study. All study participants had a small ovarian reserve (up to four follicles) and planned assisted fertilization procedures. The researchers observed an increase in the number of mature follicles in the ovaries after PRP treatments, as well as improved embryo quality and pregnancy outcome rates [36]. After a 3-month follow-up, FSH and AMH levels improved significantly in PRP-treated women, while there was no change in the control group. In addition, the overall biochemical and clinical pregnancy rates were higher in the PRP group, and the rates of first-trimester miscarriages and live births did not differ between the two groups [36].

On the other hand, the literature provides data on evaluating the ovarian PRP procedure, where no effect was found. Another meta-analysis summarized the effect of an intra-ovarian injection of PRP on patients with a poor ovarian response (POR) or premature ovarian insufficiency (POI) [37]. A separate analysis of pregnancies, AFC and AMH were performed in POI and POR groups and in age groups <35 years and >35 years. A total of 12 studies were included. There were no significant differences in POI/POR and those with <35 years or >35 years [37]. The pooled standard difference of means favoured the post-PRP injection group significantly regarding rates of embryo formation, oocyte and antral follicle counts, and AMH with low evidence. The findings stated that intraovarian injection of PRP was not associated with a significant increase in pregnancy, oocyte, embryo formation, AMH, and follicle count. Live birth rates were not calculated. No positive effect of the ovarian PRP procedure was found [37].

Overall, ovarian PRP treatment has shown more significant improvements in ovarian reserve markers and antral follicle count, which could strengthen fertility in women with reduced ovarian reserve. However, some studies [1,8,34,35,37] show no significant effects on pregnancy rates or oocyte quality, focusing attention on further challenging research to better understand the procedure’s overall effectiveness.

### 2.1. Novel Ovarian PRP Procedure Biomarkers

A diagnostic biomarker identifies the presence of a disease or condition or distinguishes a specific disease subtype, playing a crucial role in detection and classification. In the era of precision medicine, these biomarkers are advancing toward molecular and imaging-based classification systems, as seen in the evolving approach to cancer diagnosis [38]. Monitoring biomarkers are measurable indicators used to assess disease status, exposure to medical or environmental agents, or the effects of medical interventions. They are particularly significant in evaluating drug distribution, target residence time, and the extent of target modulation, as well as determining drug concentrations in body fluids to calculate appropriate dosages for desired pharmacological responses [39]. Biomarkers play a critical role in evaluating the efficiency of PRP therapy by providing measurable indicators of biological responses and therapeutic outcomes. Key biomarkers such as AMH, AFC, and GDF9 are used to monitor ovarian regeneration, follicular activation, and oocyte maturation potential following PRP treatment [40,41]. Additionally, inflammatory and angiogenic markers like vascular endothelial growth factor (VEGF) and platelet-derived growth factors (PDGF) help assess tissue repair and vascular stabilization processes initiated by PRP [34,35,42]. These biomarkers objectively assess PRP’s regenerative efficacy and guide personalized therapy adjustments.

Given the ovary’s angiogenic nature and the critical role of platelet-derived growth factors in vascular activation and stabilization, autologous PRP treatment is considered a potential facilitator of ovarian tissue regeneration. PRP, also GDF9, is a TGF-β superfamily member and biomarker of oocyte maturation, whose mutations are associated with premature ovarian dysfunction. However, the observed effects of PRP injections may result from mechanical disruption rather than growth factors, and any benefits could be temporary [43]. Zhang et al. investigated the effects of PRP and platelet-poor plasma (PPP) on bone marrow-derived mesenchymal stem cells (BM-MSCs) [44]. PRP enhanced BM-MSC proliferation, osteogenic differentiation, migration, and protection against senescence β-galactosidase, partly through activating the PI3K/AKT signalling pathway. At the same time, PPP showed limited effects [44]. PRP treatment reduced the expression of β-galactosidase, a key marker of cellular senescence, suggesting that PRP offers substantial protection against cell ageing. Additionally, PRP increased the expression of pluripotency markers (Sox2, Sall4, Oct4, and Nanog) and altered epithelial–mesenchymal transition-related proteins, highlighting its potential to promote the regenerative capabilities of BM-MSCs [44].

### 2.2. Electrochemical Biosensors as a Tool for PRP Therapy Effectiveness Evaluation

Electrochemical sensors used in the analysis and testing have several advantages over traditional analytical methods, such as ELISA and mass spectrometry, which have become standard in many laboratories (Table 2).

The advantages might be seen in Table 2. Electrochemical sensors are optimal in cost, easy to use, portable or integrated into wearable devices and mobile devices, and provide real-time monitoring, if engineered, without complex overlapping of analytes if used more than for one analysis [54].

Also, multi-parameter electrochemical data are suitable for computational methods (such as computational cluster assay) to support evaluation and for accurate discrimination between non-infected and infected samples or some overlapping, providing better accuracy in classifying plasma samples [55].

Electrochemical biosensors, unlike conventional electrochemical sensors, integrate a biological recognition element such as enzymes [56], antibodies [57], molecularly imprinted polymers [58], or aptamers [59], which provides high specificity for detecting biomolecules like platelet-derived growth factor-BB (PDGF-BB) [60], vascular endothelial growth factor (VEGF) [59], and transforming growth factor-beta (TGF-β) [61] in PRP. For instance, a study demonstrated using an electrochemical aptasensor for detecting PDGF-BB, achieving a detection limit of 18 pg/mL [62]. Similarly, another research developed an electrochemiluminescence biosensor for VEGF165 detection, with a detection limit of 0.68 pg/mL [61]. This makes them particularly suited for PRP analysis, as they enable precise, quantitative, and real-time monitoring of biomarkers essential for evaluating therapeutic potential. Unlike general electrochemical sensors, electrochemical biosensors can handle complex biological samples like PRP with minimal preparation, preserving sample integrity and reducing analysis time. Additionally, their ability to detect multiple biomarkers [63] simultaneously allows for comprehensive PRP quality and activity assessments in a single test. Miniaturized and portable designs further enhance their practicality for point-of-care diagnostics, making them an accessible, cost-effective, and reliable solution for monitoring PRP therapy and optimizing regenerative medicine outcomes [64,65].

Despite many advantages of electrochemical biosensors such as high sensitivity, rapid response time, and cost-effectiveness, they also have certain limitations compared to other diagnostic techniques. Comparing Quartz Crystal Microbalance sensors to electrochemical ones may show lower selectivity and stability in complex biological samples. In addition, fluorescence-based devices have higher multiplexing capabilities and improved spatial resolution. This is important to know while applying these methods in certain diagnostic applications. Electrochemical sensors may require frequent calibration since signal drift is impacted by electrode surface fouling.

Instrumental methods, including gas and liquid chromatography, provide high accuracy and sensitivity but are hindered by the need for expensive equipment, complex operations, and lengthy analysis times. Sensor-based analytical techniques offer a more cost-effective and faster alternative, requiring minimal training for operation. These affordable methods are increasingly valued for their ability to detect a wide range of substances efficiently [66]. Molecular imprinting involves the assembly of specific functional monomers around a template molecule, followed by polymerization with a crosslinker [67]. While physical methods enable sensitive signal transduction, achieving reliable selectivity in bioanalytical systems remains challenging, prompting the development of new chemical materials and technologies [68]. Molecularly imprinted polymers (MIPs) are synthetic polymer structures that create highly specific, selective, and sensitive analytical systems [69]. Conducting polymers are commonly employed in MIP development, often fabricated using electrochemical methods [66]. The simplicity of the electrochemical MIP manufacturing process, along with the potential to enhance their properties through dopants or adjustments to electrochemical parameters, has garnered considerable interest from researchers [70]. MIPs are highly promising materials due to their ability to create binding sites that are specifically complementary to the imprinted template molecules. Their technology emphasises binding selectivity and enables the imprinting of template molecules at room temperature without causing denaturation or conformational changes [71]. MIPs can be effectively engineered to recognize a wide range of small-molecular-weight template molecules imprinted within various polymers such as polypyrrole (Ppy), polyaniline, and others. These MIPs are applicable in analytical systems utilizing diverse analytical techniques [14,20,70].

MIPs hold significant potential for gynaecological diagnostics due to their exceptional selectivity, stability, and affordability. Biosensors provide a rapid, sensitive, and non-invasive diagnostic approach by converting biological responses into measurable signals. Incorporating MIPs into biosensors enhances their specificity and sensitivity, which is crucial for detecting low-abundance biomarkers in complex biological samples associated with gynaecological diseases [72]. For example, an MIP capable of recognizing E1 molecules was fabricated using the sol–gel method and integrated with an electrochemical luminescence sensor (MIP-ECL), combining the high sensitivity of ECL with the high selectivity of the MIP [73]. A molecularly imprinted polythioaniline sensor was created for oestradiol detection using potentiodynamic electropolymerization of PATP-functionalized AuNPs on gold electrodes, resulting in high sensitivity, selectivity, and reproducibility [73]. Another electrochemical sensor was developed for the selective and sensitive detection of the endocrine disruptor 17-β-oestradiol (E2), using a molecularly imprinted poly(p-aminophenol) composite supported by silver nanoparticles capped with 2-mercaptobenzoxazole [74]. Scientists developed a portable, smartphone-based electrochemical sensor for detecting dydrogesterone in human plasma, utilizing screen-printed gold electrodes (SPAuEs) modified with a biomimetic molecularly imprinted poly-methacrylic acid-co-methyl methacrylate [75]. A simple sensor for detecting progesterone was developed using an MIP, with gold nanoparticles electrochemically generated on glassy carbon electrodes. The sensor maintained stable performance for up to 30 days, allowing multiple consecutive measurements, and showed promise for real sample analysis, such as quantifying progesterone in calf serum, with potential for developing sensors for other non-electrochemically active analytes [76].

## 3. Discussion

The effectiveness of the ovarian PRP procedure remains an open matter of debate. Not all obstetricians and gynaecologists perform this procedure. The medical norm of the obstetrician-gynaecologist does not mention information about the provision of the PRP procedure. It is also not taught during residency in obstetrics and gynaecology. More scientifically, valid studies are needed to draw conclusions.

However, with more and more scientific articles showing the positive benefits of the ovarian PRP procedure, why is it not part of normal daily clinical practice? Why premature ovarian insufficiency is not an indication to perform ovarian PRP? If the incidence of oncological diseases is increasing, why is the ovarian PRP procedure not included in the treatment algorithms after chemotherapy?

To this day, it is still not possible to make grandiose conclusions and use the ovarian PRP procedure routinely. There is currently insufficient information on using the PRP procedure to improve ovarian function. In most of the studies, the insufficient number of included participants, or specific population, and the low precision of the protocols are prevalent without estimating the number of living offspring [77].

An analysis of scientific articles related to adverse events (ARs) associated with PRP therapy was performed on the PubMed platform. The literature revealed that the main NRs are postoperative infections, inflammation, allergic reactions, and the development of fibrotic nodules at the injection site. The most common NRs were postoperative infections. It was speculated that PRP may have been contaminated with microorganisms at some point in preparing the platelet mass. This could be because PRP cannot be sterilized like medicine. However, the specific process involved in the risk of microbial contamination remains unclear.

Recently, biosensors are playing the most significant role in biomedical analysis. Conducting polymers, such as polypyrrole, polythiophene, polyaniline, and others, are highlighted for their versatility and suitability in sensor applications [78]. The design of biosensors and chemical sensors is rapidly evolving as new materials with advanced sensing, catalytic, and charge transfer properties are used in these devices. Among them are MXenes, which are a novel class of 2D materials with metallic conductivity or semiconducting properties, making them ideal for sensors, biosensors, biofuel cells, and wearable bioelectronics [79,80]. Structurally related to graphene, MXenes primarily consist of 2D transition metal carbides, nitrides, or carbonitrides [79].

Advanced computation methods are applied to predict the most optimal designs of artificial receptors and molecularly imprinted polymers applied in biomedical analysis [81,82]. Understanding the physiological levels of biomarkers is essential for evaluating the effectiveness and applicability of electrochemical biosensors for ovarian function evaluation. While achieving high sensitivity and low detection limits is important, electrochemical biosensors must also accurately detect biomarker variations within their normal physiological range. Some biomarkers are absent under normal conditions and only emerge in pathological states, necessitating low detection limits for early detection. In contrast, others are present normally and fluctuate during disease, requiring a dynamic detection range encompassing both normal and pathological levels [83]. Therefore, electrochemical biosensors for PRP might be a great solution. Also, biosensor-based analytical techniques provide a faster, more affordable option that is easy to operate with minimal training. It might be modified and engineered to be portable and wearable, making it an attractive solution for patients and/or medical staff.

## 4. Materials and Methods

The literature selection was based on some inclusion and exclusion criteria, with studies categorized into two blocks: PRP biomarkers and electrochemistry. Inclusion criteria for PRP-focused studies required investigating biomarkers relevant to PRP treatment evaluation and prognosis, including in vitro and in vivo research published in peer-reviewed journals, with clear data on study design, participant details, and outcomes. Exclusion criteria excluded studies lacking a focus on PRP biomarkers, non-peer-reviewed articles, and those with incomplete or unclear data. For electrochemical methods, inclusion criteria targeted studies on molecularly imprinted polymers (MIPs), their development, strategies, and applications in ovarian diagnostics, requiring peer-reviewed publication and comprehensive data. Excluded were studies not emphasising electrochemical approaches in ovarian diagnostics, non-peer-reviewed articles, and those with insufficient or ambiguous information.

A literature search was performed using one of the best-recognised PubMed databases, employing a combination of keywords such as “platelet-rich plasma”, “biomarkers”, “electrochemistry”, “molecularly imprinted polymers”, “diagnosis”, “prognosis”, and “ovarian”. Boolean operators (AND, OR) were used to refine and optimize the search. The focus was on studies published up to 2024, emphasising clinical research articles from 2021 to 2024.

## 5. Conclusions

The ovarian PRP procedure is an innovative technique that can hopefully help preserve ovarian function. There is evidence that this procedure can stimulate natural regeneration processes, promote ovarian tissue health, and possibly improve fertility.

Given the small profile of the studies performed, the hypothesized therapeutic benefit of the ovarian PRP procedure must be investigated in larger, well-controlled studies. Currently, there are no standardized models and guidelines for applying the ovarian PRP procedure to improve a woman’s fertility and maintain a high ovarian reserve. Also, novel biomarkers and biosensors could lead to a better understanding of how the procedure affects ovarian tissue. Electrochemical biosensors, integrating biological recognition elements like enzymes, antibodies, or aptamers, offer high specificity and sensitivity for detecting biomarkers such as PDGF-BB, VEGF, and TGF-β in PRP, enabling precise and real-time monitoring essential for evaluating therapeutic potential. Their ability to handle complex biological samples with minimal preparation detect multiple biomarkers simultaneously, and their portability makes them ideal for point-of-care diagnostics and optimizing regenerative medicine outcomes.

PRP injections into the ovaries can become an alternative or holistic medicine for treating ovarian insufficiency, infertility, and reproductive health problems, as the procedure utilizes the body’s own healing capabilities. Maintaining the balance of a woman’s sex hormones ensures not only the reproductive capabilities of the body but also reduces the symptoms of perimenopause and the risk of osteoporosis and improves a woman’s psychological health. Further research is needed to understand the benefits of ovarian PRP therapy and its connection with novel biomarkers and biosensors.

## Figures and Tables

**Table 1:** Seven major growth factors found in platelets.

Growth Factor Name	Biological Activity	Role of Ovarian Function
Platelet-derived growth factor (PDGF)	It is a cell division stimulator.	Participates in the regulation of the ovary gland in an autocrine and paracrine manner and has the pathological effect of promoting the excessive growth of related tissue cells [26].
Transforming growth factors (TGFs)	Divided into two types: TGFα and TGFβ. They participate in the healing process of fractures by stimulating osteogenesis and the synthesis of mature osteocytes.	TGF-β activity suppresses cellular growth and promotes differentiation [27].
Vascular endothelial growth factor (VEGF)	Stimulates the genesis of new blood vessels during embryonic development and participates in the formation of bypass vessels in case of vascular obstruction.	Plays a role in the cyclic growth of ovarian follicles and corpus luteum development and maintenance, mediating ovarian angiogenesis [28].
Epidermal growth factor (EGF)	One of the most important proteins of skin growth factor. It participates in the production of collagen, elastin, and hyaluronic acid.	Stimulates oocyte maturation in a variety of mammalian and non-mammalian species [26].
Fibroblast growth factor (FGF)	FGF proteins are mitogens involved in both normal growth and wound healing.	Alters the growth and differentiation of reproductive tissues [29].
Connective tissue growth factor (CTGF)	Stimulates cell proliferation, migration, adhesion, survival, differentiation, and the synthesis of extracellular matrix proteins.	It is required for normal follicle development and ovulation [30].
Insulin-like growth factor (IGF)	This factor stimulates protein synthesis.	Acts as an amplifier to the hormonal action of gonadotropins [26].

**Table 2:** Comparison of analytical methods with electrochemical sensors.

Feature	Electrochemical Sensors	ELISA Method	Mass Spectrometry	Refs.
Cost	Low	Moderate to high	Very high	[45,46]
Speed	High	Moderate	Low	[47,48]
Portability	High	Moderate to low	Very low	[49]
Volume (sample)	Minimal	Moderate	Moderate	[47,48]
Real-time monitoring	Easily adoptable	Moderate to adopt	Moderate to adopt	[50]
Complexity/overlapping	Feasible	Limited	Limited	[51,52,53]

## Data Availability

Data sharing does not apply to this article as no datasets were generated.

## Abbreviations

The following abbreviations are used in this manuscript:

AMH	Anti-Müllerian hormone
BM-MSCs	Bone marrow-derived mesenchymal stem cells
CTGF	Connective tissue growth factor
EGF	Epidermal growth factor
ESHRE	The European Society of Reproductive Medicine
FGF	Fibroblast growth factor
FSH	Follicle-stimulating hormone
IGF	Insulin-like growth factor
LH	Luteinizing hormone
MIP	Molecularly imprinted polymers
PDGF	Platelet-derived growth factor
PDGF-BB	Platelet-derived growth factor-BB
POI	Premature ovarian insufficiency
POR	Poor ovarian response
PPP	Platelet-poor plasma
PRP	Platelet-rich plasma
TGF	Transforming growth factor
VEGF	Vascular endothelial growth factor
